# Maternal age extremes and adverse pregnancy outcomes in low-resourced settings

**DOI:** 10.3389/fgwh.2023.1201037

**Published:** 2023-11-28

**Authors:** Paul Nyongesa, Osayame A. Ekhaguere, Irene Marete, Constance Tenge, Milsort Kemoi, Carla M. Bann, Sherri L. Bucher, Archana B. Patel, Patricia L. Hibberd, Farnaz Naqvi, Sarah Saleem, Robert L. Goldenberg, Shivaprasad S. Goudar, Richard J. Derman, Nancy F. Krebs, Ana Garces, Elwyn Chomba, Waldemar A. Carlo, Musaku Mwenechanya, Adrien Lokangaka, Antoinette K. Tshefu, Melissa Bauserman, Marion Koso-Thomas, Janet L. Moore, Elizabeth M. McClure, Edward A. Liechty, Fabian Esamai

**Affiliations:** ^1^ Department of Obstetrics and Gynecology, Moi University School of Medicine, Eldoret, Kenya; ^2^ Division of Neonatal-Perinatal Medicine, Indiana University School of Medicine, Indiana University, Indianapolis, IN, United States; ^3^ Department of Child Health and Paediatrics, Moi University School of Medicine, Eldoret, Kenya; ^4^ Social Statistical, and Environmental Sciences Unit, RTI International, Durham, NC, United States; ^5^ Department of Community and Global Health, Richard M. Fairbanks School of Public Health, IU-Indianapolis, Indianapolis, IN, United States; ^6^ Department of Pediatrics, Datta Meghe Institute of Medical Sciences, Wardha, Maharashtra, India; ^7^ Department of Global Health, Boston University School of Public Health, Boston, MA, United States; ^8^ Department of Community Health Sciences, Aga Khan University, Karachi, Pakistan; ^9^ Department of Obstetrics and Gynecology, Columbia University School of Medicine, New York, NY, United States; ^10^ Women's and Children's Health Research Unit, J N Medical College Belagavi, KLE Academy Higher Education and Research, Karnataka, India; ^11^ Global Affairs, Thomas Jefferson University, Philadelphia, PA, United States; ^12^ Department of Pediatrics, University of Colorado School of Medicine, Denver, CO, United States; ^13^ Department of Pediatrics, Instituto de Nutrición de Centroamérica y Panamá, Guatemala City, Guatemala; ^14^ Department of Pediatrics, University Teaching Hospital, Lusaka, Zambia; ^15^ Department of Pediatrics, University of Alabama at Birmingham, Birmingham, AL, United States; ^16^ Department of Pediatrics, Kinshasa School of Public Health, Kinshasa, Democratic Republic of Congo; ^17^ School of Public Health, University of Kinshasa, Kinshasa, Democratic Republic of Congo; ^18^ Department of Pediatrics, University of North Carolina at Chapel Hill, Chapel Hill, NC, United States; ^19^ Eunice Kennedy Shriver National Institute of Child Health and Human Development, National Institutes of Health, Bethesda, MA, United States

**Keywords:** pregnancy outcomes, low-and middle-income country, adolescent pregnancy, advanced maternal age pregnancy, maternal mortality ratio, neonatal mortality

## Abstract

**Introduction:**

Adolescent (<20 years) and advanced maternal age (>35 years) pregnancies carry adverse risks and warrant a critical review in low- and middle-income countries where the burden of adverse pregnancy outcomes is highest.

**Objective:**

To describe the prevalence and adverse pregnancy (maternal, perinatal, and neonatal) outcomes associated with extremes of maternal age across six countries.

**Patients and methods:**

We performed a historical cohort analysis on prospectively collected data from a population-based cohort study conducted in the Democratic Republic of Congo, Guatemala, India, Kenya, Pakistan, and Zambia between 2010 and 2020. We included pregnant women and their neonates. We describe the prevalence and adverse pregnancy outcomes associated with pregnancies in these maternal age groups (<20, 20–24, 25–29, 30–35, and >35 years). Relative risks and 95% confidence intervals of each adverse pregnancy outcome comparing each maternal age group to the reference group of 20–24 years were obtained by fitting a Poisson model adjusting for site, maternal age, parity, multiple gestations, maternal education, antenatal care, and delivery location. Analysis by region was also performed.

**Results:**

We analyzed 602,884 deliveries; 13% (78,584) were adolescents, and 5% (28,677) were advanced maternal age (AMA). The overall maternal mortality ratio (MMR) was 147 deaths per 100,000 live births and increased with advancing maternal age: 83 in the adolescent and 298 in the AMA group. The AMA groups had the highest MMR in all regions. Adolescent pregnancy was associated with an adjusted relative risk (aRR) of 1.07 (1.02–1.11) for perinatal mortality and 1.13 (1.06–1.19) for neonatal mortality. In contrast, AMA was associated with an aRR of 2.55 (1.81 to 3.59) for maternal mortality, 1.58 (1.49–1.67) for perinatal mortality, and 1.30 (1.20–1.41) for neonatal mortality, compared to pregnancy in women 20–24 years. This pattern was overall similar in all regions, even in the <18 and 18–19 age groups.

**Conclusion:**

The maternal mortality ratio in the LMICs assessed is high and increased with advancing maternal age groups. While less prevalent, AMA was associated with a higher risk of adverse maternal mortality and, like adolescence, was associated with adverse perinatal mortality with little regional variation.

## Introduction

Adverse pregnancy outcomes such as maternal, perinatal, and neonatal mortality are essential global, regional, and national health indicators (1–3). All pregnancies carry risks. However, women ≤19 (adolescents) and those ≥35 years of age [advanced maternal age (AMA)] have been associated with a higher risk of adverse pregnancy outcomes, including maternal mortality, stillbirth, perinatal, neonatal, infant, and under-five mortality (4–8). Complications during childbirth are a leading cause of adolescent deaths (9). Likewise, AMA pregnancies are associated with a higher prevalence of morbidities such as diabetes, hypertension, and obesity, known to exacerbate adverse pregnancy outcomes (10–12).

The low- and middle-income countries (LMICs) of Africa and South Asia account for 87% of global maternal deaths (3). These regions also account for 95% of the global adolescent pregnancy burden—translating into 21 million yearly pregnancies in girls aged 15–19 (8). Available evidence indicates a rising trend in AMA pregnancies (13–15). However, these reports mainly originate from high-income countries; similar data from LMICs are sparse, often single-centered (16–20), historical cohorts (21), cross-sectional facility-based or demographic health surveys (6).

To appraise the impact of existing and inform future health policies, current and generalizable data on outcomes of adolescents and AMA pregnancies from LMICs are needed. This knowledge is critical to understanding local, national, and global progress towards the 2030 sustainable development goals of improving pregnancy outcomes (2). Hence, this study aimed to describe adolescent and AMA pregnancy rates and their associated adverse pregnancy outcomes in six LMICs participating in a large prospective maternal and newborn birth registry.

## Patients and methods

We performed a historical cohort study on prospectively collected data from the Global Network for Women's and Children's Health Research Maternal and Neonatal Health Registry (global network registry). The global network registry is a multicountry prospective, population-based observational study that monitors all pregnant women and their pregnancy outcomes in seven sites within six LMICs (22, 24). The countries include the Democratic Republic of Congo (North and South Ubangi Provinces); Guatemala (Western Highlands); India (Belagavi and Nagpur); Kenya (Western region); Pakistan (Thatta, a rural district of Sindh province, near the city of Karachi); and Zambia (south and east of the capital city of Lusaka). The study population includes both peri-urban and rural settings. A previous publication details the overall purpose, methods, and data collection techniques of the global network registry (23).

For this study, we included all women enrolled in the registry between January 2010 and December 2020. The Democratic Republic of Congo began participation in the registry in mid-2013. We excluded women lost to follow-up before delivery, those who had a spontaneous or medically induced abortion or other pregnancy loss <20 weeks, medically terminated pregnancy at any point, women who gave birth to infants weighing less than 500 g, and those with no maternal age recorded. For this study, we excluded deliveries that were <500 g (defined as lower cut-off for stillbirths) because the majority of women we enrolled were at 20 weeks or greater (i.e., > 500 g).

This study evaluated maternal, perinatal, and neonatal outcomes. The maternal outcomes included antepartum and postpartum hemorrhage, obstructed labor, hypertensive disorders, sepsis, and maternal mortality within 42 days postpartum. The perinatal and neonatal outcomes included preterm birth (live birth at <37 completed weeks' gestation), low birthweight (live birth weighing <2,500 g at birth), stillbirth [deaths occurring in fetuses >500 g (or >22 completed weeks gestation) noted at delivery], early neonatal deaths (neonatal deaths that occur 0–7 days after birth), neonatal mortality (neonatal deaths 0–28 days after birth), perinatal deaths (early neonatal deaths and stillbirths combined). Most women enrolled after 20 weeks gestation; consequently, we did not have data on early miscarriage/spontaneous abortions. Thus, we did not include these adverse pregnancy outcomes.

### Statistical analysis

Maternal and infant demographic characteristics and clinical outcomes were compared using standard descriptive statistics stratified by maternal age categories (<20, 20–24, 25–29, 30–35, and >35 years). We chose this categorization because the South Asian regions did not enroll participants younger than 18. We also present maternal and infant demographic characteristics and clinical outcomes by WHO country region (Africa, Southeast Asia, and Central America). Given the large sample size, we do not report *p*-values for the demographic and clinical comparison because even minimal differences become statistically significant.

To estimate the association of maternal age with maternal and perinatal outcomes, we used a Poisson model for each outcome for the entire cohort and within each region. We obtained point and interval estimates of the relative risk associated with maternal age groups (<20, 25–29, 30–35, and >35 years) from Poisson models controlling for site, parity, multiple gestation, maternal education (any or none), antenatal care and delivery location compared to mothers aged 20–24 years, consistent with a prior global network publication. Poisson models were used to evaluate if the effect of maternal age on maternal, perinatal, and neonatal mortality is modified by education, attendance to at least one antenatal visit, and delivery location. Relative risks, 95% confidence intervals, and interaction *p*-values are obtained from a Poisson model for each outcome, including the covariates above and two-way interactions between maternal age and education, antenatal care, and delivery location. We performed regional analysis with further age categorization to <18 and 18–19 for the subgroups of women from the African region and Guatemala. All data were analyzed using SAS version 9.4 (SAS Institute, Cary, NC, USA).

## Results

### Description of the study population

**Figure 1:**
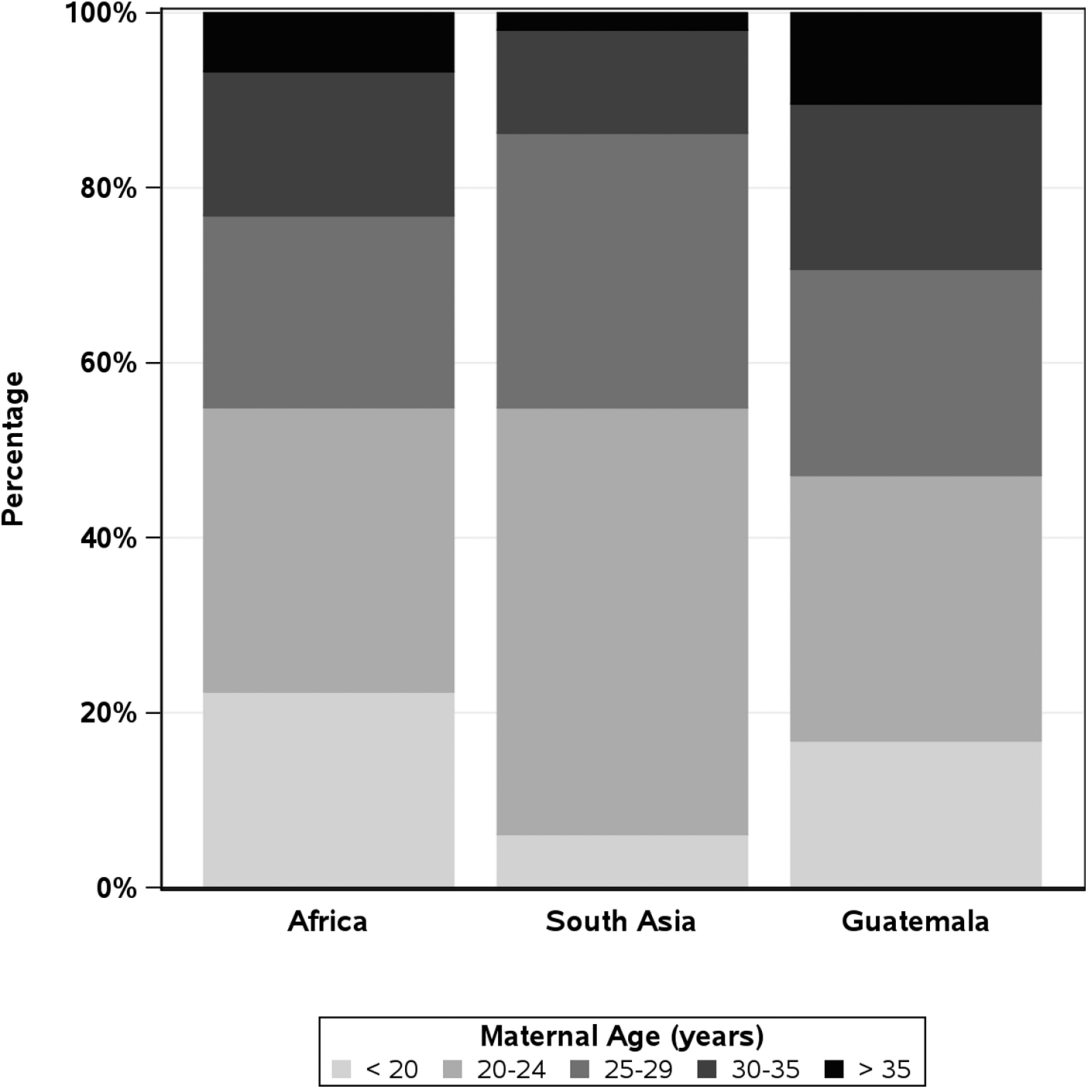
Maternal age distribution by global network region.

Over the 11-year study period, 644,709 women were screened. Of these, 602,884 delivered 608,918 babies who met the inclusion criteria and were analyzed (Table 1). Women aged 20–24 years accounted for 41% (245,289) of deliveries, while adolescent and AMA deliveries accounted for 13% (78,584) and 5% (28,677), respectively (Table 2). By geographic regions, the African region had the highest rates of adolescents (22%), Guatemala had the highest rates of AMA pregnancies (10%), and Asia had the lowest rates of adolescent (6%) and AMA (2%) pregnancies (Figure 1).

**Table 1:** Distribution of the included and excluded women and neonates by study country.

Consort diagram information	Overall	DRC^c^	Zambia	Guatemala	Belagavi	Pakistan	Nagpur	Kenya
Screened, *n*	644,709	43,897	71,648	94,130	142,315	110,878	94,844	86,997
Ineligible, *n* (%)	2,543 (0.4)	0 (0.0)	2 (0.0)	72 (0.1)	1 (0.0)	2,443 (2.2)	1 (0.0)	24 (0.0)
Eligible, *n* (%)	642,166 (99.6)	43,897 (100.0)	71,646 (100.0)	94,058 (99.9)	142,314 (100.0)	108,435 (97.8)	94,843 (100.0)	86,973 (100.0)
Did not consent, *n* (%)	1,118 (0.2)	0 (0.0)	0 (0.0)	1,010 (1.1)	24 (0.0)	81 (0.1)	0 (0.0)	3 (0.0)
Consented, *n* (%)	641,048 (99.8)	43,897 (100.0)	71,646 (100.0)	93,048 (98.9)	142,290 (100.0)	108,354 (99.9)	94,843 (100.0)	86,970 (100.0)
Lost to follow-up prior to delivery, *n* (%)	7,279 (1.1)	616 (1.4)	410 (0.6)	950 (1.0)	67 (0.0)	2,218 (2.0)	386 (0.4)	2,632 (3.0)
Delivered, *n* (%)	633,769 (98.9)	43,281 (98.6)	71,236 (99.4)	92,098 (99.0)	142,223 (100.0)	106,136 (98.0)	94,457 (99.6)	84,338 (97.0)
Exclusions, *n* (%)	30,885 (4.9)	554 (1.3)	686 (1.0)	894 (1.0)	14,160 (10.0)	7,736 (7.3)	5,833 (6.2)	1,022 (1.2)
Gestational at delivery < 20 weeksks^a^	28,113	448	608	828	13,536	7,077	5,230	386
Medically terminated. pregnancy (MTP)	1,597	51	12	2	482	476	545	29
Birthweight < 500 g	189	24	11	48	64	8	29	5
Maternal age missing	986	31	55	16	78	175	29	602
Deliveries included *n*	602,884	42,727	70,550	91,204	128,063	98,400	88,624	83,316
Births included^b^, *n*	608,918	43,479	71,258	91,846	129,023	99,553	89,340	84,419

^a^ Includes miscarriages with gestational age missing.

^b^ All MNH Registry 2010–2020 births excluding deliveries <20 weeks gestation, medically terminated pregnancies, infants with.

measured birthweight < 500 g and participants missing maternal age.

^c^ DRC began participation in the MNH registry in mid-2013, so it does not have data for 2010–2013 births.

**Table 2:** Maternal demographics and health care utilization by age group.

Characteristic	Overall	Maternal age category				
		<20	20–24	25–29	30–35	>35
Mothers, *n* (%)	602,884	78,584 (13.0)	245,289 (40.7)	163,583 (27.1)	86,751 (14.4)	28,677 (4.8)
Maternal education, *n* (%)	601,925	78,486	244,802	163,309	86,681	28,647
No formal schooling	143,096 (23.8)	7,531 (9.6)	36,011 (14.7)	51,264 (31.4)	37,098 (42.8)	11,192 (39.1)
Primary or secondary	417,199 (69.3)	69,319 (88.3)	187,679 (76.7)	98,311 (60.2)	45,211 (52.2)	16,679 (58.2)
University +	41,630 (6.9)	1,636 (2.1)	21,112 (8.6)	13,734 (8.4)	4,372 (5.0)	776 (2.7)
Parity, *n* (%)	600,128	78,023	243,837	162,953	86,646	28,669
0	193,940 (32.3)	63,268 (81.1)	104,902 (43.0)	21,623 (13.3)	3,687 (4.3)	460 (1.6)
1–2	251,347 (41.9)	14,361 (18.4)	125,094 (51.3)	88,733 (54.5)	20,568 (23.7)	2,591 (9.0)
3 +	154,841 (25.8)	394 (0.5)	13,841 (5.7)	52,597 (32.3)	62,391 (72.0)	25,618 (89.4)
Multiple births, *n* (%)	5,872 (1.0)	443 (0.6)	1,929 (0.8)	1,751 (1.1)	1,311 (1.5)	438 (1.5)
Body mass index (BMI) measured^a^ (Kg/m^2^), *n* (%)	495,160 (95.3)	56,376 (93.6)	206,116 (96.0)	139,086 (96.1)	70,857 (94.7)	22,725 (90.8)
Mean (std)	21.7 (4.0)	21.7 (3.3)	20.9 (3.5)	21.8 (4.0)	23.1 (4.6)	24.6 (4.9)
Median (min-max)	21.2 (12–65)	21.5 (12–63)	20.5 (12–64)	21.1 (12–60)	22.4 (12–60)	23.9 (13–65)
At least one antenatal care (ANC) visit, *n* (%)	585,029 (97.1)	77,418 (98.6)	241,290 (98.5)	157,370 (96.3)	81,838 (94.4)	27,113 (94.6)
At least four antenatal care visits, *n*/*N* (%)	283,745/482,920 (58.8)	37,887/64,492 (58.7)	123,189/189,331 (65.1)	75,968/132,124 (57.5)	35,264/72,595 (48.6)	11,437/24,378 (46.9)
Trimester of first ANC visit, *n* (%)	544,102	74,097	227,931	143,647	73,479	24,948
First (0–14 weeks)	241,148 (44.3)	25,897 (35.0)	122,945 (53.9)	65,239 (45.4)	21,817 (29.7)	5,250 (21.0)
Second (15–28 weeks)	240,743 (44.2)	40,479 (54.6)	85,358 (37.4)	60,734 (42.3)	39,138 (53.3)	15,034 (60.3)
Third (29–42 weeks)	62,211 (11.4)	7,721 (10.4)	19,628 (8.6)	17,674 (12.3)	12,524 (17.0)	4,664 (18.7)
Delivery attendant, *n* (%)	602,599	78,560	245,198	163,501	86,678	28,662
Physician	212,623 (35.3)	21,342 (27.2)	104,811 (42.7)	57,692 (35.3)	21,919 (25.3)	6,859 (23.9)
Nurse/nurse midwife/ Health worker	232,319 (38.6)	37,846 (48.2)	95,416 (38.9)	59,045 (36.1)	30,231 (34.9)	9,781 (34.1)
Traditional birth attendant	126,502 (21.0)	15,579 (19.8)	35,246 (14.4)	37,487 (22.9)	28,283 (32.6)	9,907 (34.6)
Family/self/other	31,155 (5.2)	3,793 (4.8)	9,725 (4.0)	9,277 (5.7)	6,245 (7.2)	2,115 (7.4)
Delivery location, *n* (%)	602,592	78,555	245,184	163,503	86,689	28,661
Hospital	261,466 (43.4)	30,316 (38.6)	125,046 (51.0)	68,594 (42.0)	28,394 (32.8)	9,116 (31.8)
Clinic/health center	193,592 (32.1)	29,269 (37.3)	76,266 (31.1)	52,432 (32.1)	27,428 (31.6)	8,197 (28.6)
Home/other	147,534 (24.5)	18,970 (24.1)	43,872 (17.9)	42,477 (26.0)	30,867 (35.6)	11,348 (39.6)
Placed on mother's chest after delivery or skin to skin, *n* (%)	313,126 (53.2)	48,742 (63.5)	136,994 (57.1)	76,541 (47.9)	37,186 (44.1)	13,663 (49.5)

^a^ Kenya did not consistently obtain height measurements; therefore, Kenyan BMI data is excluded.

The demographic differences between age groups are presented in Table 2. The proportion without education was lowest in adolescents (10%) and highest in AMA's (39%) and increased with advancing maternal age groups. The proportion of pregnancies that resulted in multiple births and parity of ≥3 increased with increasing age groups. The mean body mass index was 22 kg/m^2^ in adolescents, 21 kg/m^2^ in the 20–24-year group, and 25 kg/m^2^ in the AMA group. Any antenatal visit attendance declined with advancing age groups. The use of traditional birth attendants decreased between the <20 (20%) and 20–24 age group (14%) and increased with subsequent age groups 25–29 (23%), 30–35 (33%), and >35 years (35%). This pattern was similar for home births. Hospital or health care center deliveries were highest in the 20–24 age group (82%), followed by the adolescent group (76%), and declined with AMA to 60%.

### Frequencies of adverse maternal outcomes by age group

The maternal, perinatal, and neonatal outcomes are presented in Table 3. The overall maternal mortality ratio (MMR) was 147 deaths per 100,000 live births. The MMR was 132 in the African region, 172 in the South Asian region, and 98 in Guatemala (Supplementary Tables S2, S4, S6). By maternal age group, MMR increased with advancing maternal age: 83 in adolescents, 111 in the 20–24 group, and 298 in the AMA group. In the African region, MMR was 91 in the <18 years group, 65 in the 18–19 years group, and highest at 319 in the AMA group (Supplementary Table S2). In the South Asian region, MMR was lowest at 112 in adolescents and highest at 399 in the AMA group. In Guatemala, women <18 had the lowest MMR at 47, and the AMA groups had the highest at 206 deaths per 100,000 live births.

**Table 3:** Maternal and perinatal adverse outcomes by maternal age group.

Characteristic	Overall	Maternal age category				
		<20	<20	<20	<20	<20
*Mothers, n*	602,884	78,584	245,289	163,583	86,751	28,677
Obstructed/prolonged labor/failure to progress, *n* (%)	49,176 (8.2)	6,198 (7.9)	21,635 (8.8)	12,977 (7.9)	6,485 (7.5)	1,881 (6.6)
Antepartum hemorrhage, *n* (%)	7,418 (1.2)	799 (1.0)	2,077 (0.8)	2,247 (1.4)	1,701 (2.0)	594 (2.1)
Postpartum hemorrhage, *n* (%)	10,899 (1.8)	1,277 (1.6)	3,098 (1.3)	3,252 (2.0)	2,432 (2.8)	840 (2.9)
Evidence of hypertensive disease/severe pre-eclampsia/ eclampsia, *n* (%)	15,988 (2.7)	1,525 (1.9)	5,794 (2.4)	4,304 (2.6)	3,110 (3.6)	1,255 (4.4)
Abnormal lie: breech, transverse, or oblique, *n* (%)	12,294 (2.0)	1,272 (1.6)	4,534 (1.9)	3,409 (2.1)	2,205 (2.5)	874 (3.1)
Severe infection at follow-up, *n* (%)	2,614 (0.5)	294 (0.4)	780 (0.3)	762 (0.5)	563 (0.7)	215 (0.8)
Cesarean delivery, *n* (%)	83,680 (13.9)	7,966 (10.1)	38,052 (15.5)	24,224 (14.8)	10,325 (11.9)	3,113 (10.9)
Maternal death < 42 days,n (rate/100,000 live births)	872 (147)	64 (83)	269 (111)	250 (156)	206 (243)	83 (298)
*Infants, N*	608,918	79,038	247,250	165,375	88,115	29,140
Stillbirth, *n* (rate/1,000)	16,742 (27.5)	1,869 (23.7)	5,659 (22.9)	4,709 (28.5)	3,198 (36.3)	1,307 (44.9)
Stillbirth type, *n* (%)	15,589	1,792	5,315	4,297	2,942	1,243
Macerated	4,984 (32.0)	528 (29.5)	1,656 (31.2)	1,382 (32.2)	1,005 (34.2)	413 (33.2)
Fresh	10,605 (68.0)	1,264 (70.5)	3,659 (68.8)	2,915 (67.8)	1,937 (65.8)	830 (66.8)
Neonatal death < 7 days, *n* (rate/1,000)	11,556 (19.6)	1,496 (19.5)	4,283 (17.8)	3,093 (19.3)	1,995 (23.6)	689 (24.9)
Neonatal death < 28 days, *n* (rate/1,000)	14,527 (24.6)	1,826 (23.7)	5,303 (22.0)	3,923 (24.5)	2,560 (30.3)	915 (33.0)
Perinatal mortality, *n* (rate/1,000)	28,298 (46.6)	3,365 (42.7)	9,942 (40.3)	7,802 (47.4)	5,193 (59.2)	1,996 (68.8)
Preterm birth, *n* (%)	81,105 (13.7)	11,970 (15.5)	29,504 (12.2)	22,042 (13.7)	13,208 (15.4)	4,381 (15.5)
Low birthweight (<2,500 g), *n* (%)	88,516 (14.6)	11,320 (14.3)	36,979 (15.0)	23,668 (14.4)	12,463 (14.2)	4,086 (14.1)

### Frequencies of adverse perinatal and neonatal outcomes by age group

For the entire cohort, perinatal and neonatal mortality decreased slightly between adolescents (43 and 24 deaths per 1,000 live births) and mothers aged 20–24 (40 and 22 deaths per 1,000 live births). It increased in subsequent groups, with AMA having the highest rates (69 and 33 deaths per 1,000 live births). This pattern was similar by region, except in the African region, where women <18 years had the highest neonatal mortality rates (Supplementary Tables S2, S4, S6).

### Association of risk for maternal adverse outcomes

**Figure 2:**
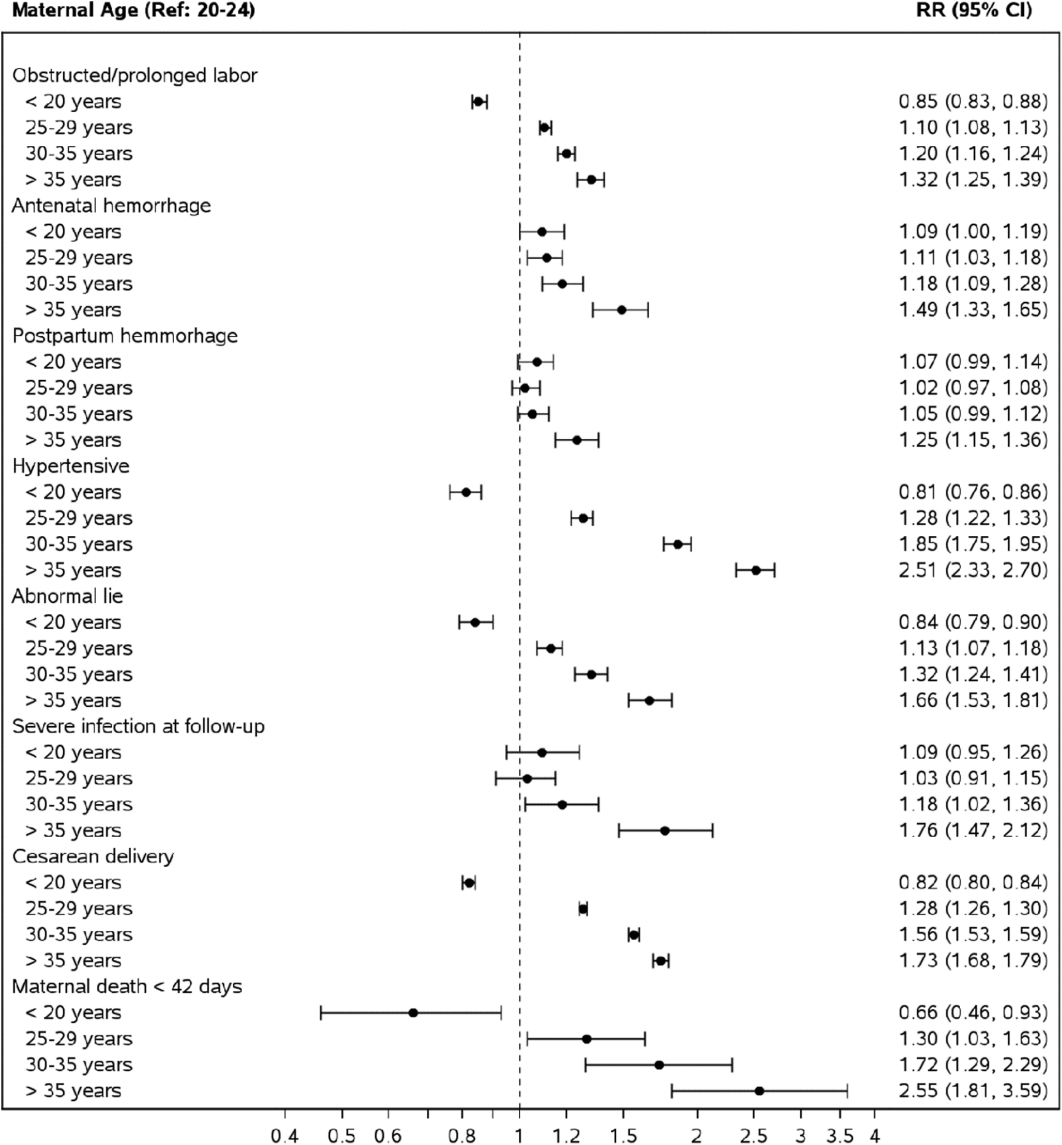
Risk of adverse maternal outcomes by maternal age groups compared to mothers aged 20–24. *Relative risks from a Poisson model adjusting for maternal age, site, parity, multiple gestation, maternal education (some or none), ANC care and delivery location.

Estimated relative risks for maternal outcomes, controlling for site, parity, multiple gestation, maternal education (any or none), antenatal care, and delivery location, are presented in Figure 2. Overall, compared to women aged 20–24, pregnancy during adolescence had a lower or no associated risk of developing any maternal morbidity we assessed. However, AMA was associated with an adjusted risk ratio (aRR) of 2.55 [95% confidence interval (CI) 1.81–3.59] for maternal mortality, 1.32 (CI 1.25–1.39) for obstructed or prolonged labor, 1.49 (CI 1.33–1.65) for antenatal hemorrhage, 1.25 (CI.15–1.36) for post-partum hemorrhage, and 1.73 (CI 1.68–1.79) for cesarean delivery. This pattern of a lower associated risk of maternal morbidity in adolescents and higher aRR in AMA compared to women aged 20–24 was observed in the South Asian region and Guatemala (Supplementary Figures S3, S5). In the African region, adolescence was associated with an increased aRR of obstructed labor, antepartum, post-partum hemorrhage, and severe infection but a lower risk of cesarean section and no difference in risk of maternal mortality compared to women aged 20–24. However, AMA showed increased aRR of all evaluated maternal morbidities except post-partum hemorrhage (Supplementary Figure S1).

### Association of risk for perinatal and neonatal adverse outcomes

**Figure 3:**
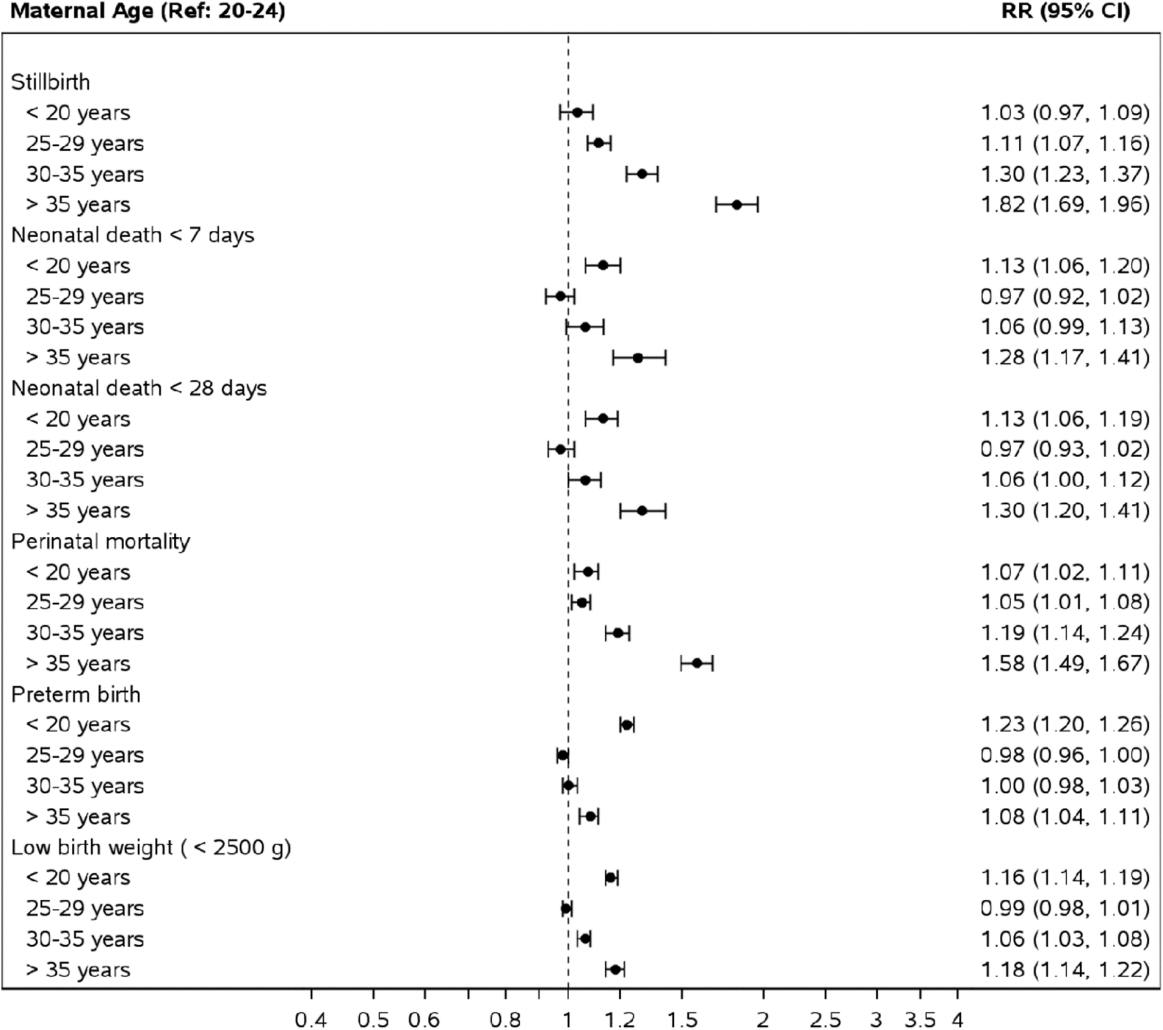
Risk of perinatal and neonatal outcomes by maternal age groups compared to mothers aged 20–24. *Relative risks obtained from a Poisson model adjusting for maternal age, site, parity, multiple gestation, maternal education (some or none), ANC care and delivery location.

For the adverse perinatal and neonatal outcomes, compared to women 20–24 years, adolescence was associated with an aRR of 1.07 (CI 1.07–1.11) for perinatal mortality, 1.13 (CI 1.06–1.19) for neonatal deaths, and 1.16, (CI 1.14–1.19) for low birth weight (Figure 3). AMA was associated with an aRR of 1.58 (CI: 1.49–1.67) for perinatal mortality, 1.30 (1.20–1.14) for neonatal mortality, and 1.18 (CI 1.14–1.22) for low birth weight status.

In the African region, the pattern of associated risk took a C-shaped pattern on the forest plot, with adolescent and AMA groups both carrying an increased associated risk of perinatal, neonatal mortality and low birth weight status compared to women aged 20–24 years (Supplementary Figure S1). In the Asian region and Guatemala, adolescents were only associated with an increased aRR of low birth weight status. In contrast, AMA was associated with perinatal, neonatal, and low birth weight status (Supplementary Figures S3, S5).

### Interaction between key variables and adverse perinatal and neonatal outcomes

In the *post-hoc* analysis, we added interaction terms to the Poisson models to evaluate if the effect of maternal age on maternal, perinatal, and neonatal mortality is modified by education (any vs. none), attendance to at least one antenatal visit or delivery location. The model for maternal mortality did not have any significant interaction terms, so it is omitted. The effect of age on perinatal mortality is modified by education (*p* < 0.001), antenatal care (*p* < 0.001) and delivery location (*p* < 0.001) (Supplementary Figure S7). The risk of perinatal mortality is higher for adolescent and AMA women compared to women aged 20–24 for women with no schooling compared to those with schooling. AMA women have an increased risk of perinatal mortality for women with and without antenatal care, while adolescent women have increased risk without antenatal care but no difference compared to women with antenatal care aged 20–24.

## Discussion

Using a robust population-based cohort spanning 11 years, we evaluated the prevalence and adverse outcomes associated with extremes of maternal age. Adolescent and AMA pregnancies represented 13% and 5% of all births, respectively. The African region had the highest proportion of adolescent pregnancies (22%), and Guatemala had the highest number of AMA pregnancies (10%). The MMR for the entire cohort was 147 deaths per 100,000 live births, increasing with advancing maternal age groupings. Adolescent pregnancy was shown to have a protective association with many maternal adverse outcomes but not perinatal or neonatal adverse outcomes. However, AMA was associated with an increased risk of adverse pregnancy outcomes. With a few exceptions, this pattern was similar in the African, Asian, and Central American regions.

In the past two decades, global MMR declined by 34% (743 to 223 deaths per 100,000 live births) (3). However, as with our findings, variations in MMR within and between global and regional geographies exist. In a large maternal cohort study involving eight countries (five from the current study) that evaluated 269,630 pregnant women, the MMR was 317 (24)—almost twice our findings. Socioeconomic differences do not explain these differences, as both studies recruited patients from rural and peri-urban communities in the African and South Asian regions. Differences in population sampling may explain the observed differences, as even within the referenced study, considerable variations existed within individual participating countries.

While we did not specifically study changes in MMR over time—as it was not the objective of this study—our finding closely mirrors data from the 2010 to 2018 global network cohort with an MMR of 157 per 100,000 live births (25). The slight difference in MMR is unlikely explained by improved health-seeking behaviors or socioeconomic status, as they were comparable between the 2010–2018 and 2020 cohorts.

We observed that overall and within regions, adolescents had the lowest maternal mortality compared to the older age groups and a lower associated risk of the adverse maternal pregnancy outcomes we evaluated. Our findings are similar to prior publications from the global network and a US-based cohort study (25, 26). Better health-seeking behaviors in adolescents may explain these findings. In our cohort, adolescents had the highest proportion of ≥1ANC visit, the second-highest proportion of first-trimester antenatal visits, ≥4 antenatal visits, and the use of skilled health personnel during delivery. These proportions consistently declined with each advancing age group higher than the 20–24 age group.

Conversely, adolescent pregnancy was associated with increased perinatal and neonatal mortality risk and low birth weight status. However, the morbidities and risk sizes we observed vary from those reported in published studies. In a study that utilized demographic and health surveys from 2004 to 2018 for Sub-Saharan and South Asian countries, the authors segregated adolescents into age groups <16, 16–7, and 18–19 and compared them with women 23–25 years (5). The authors found that all adolescent groups had increased odds of stillbirth and neonatal death in both regions (5). In our cohort, however, overall adolescent pregnancy (<20 years) was not associated with an increased risk of stillbirth. In the current study, in the African region and Guatemala, only adolescents <18 years had an increased associated risk of stillbirths. There was no increased risk of neonatal mortality in women <18 or between 18 and 19 years in Guatemala or the <20 age group in the Asian region. However, both adolescent groups in the African region had an increased associated risk of neonatal mortality. This pattern of lower maternal but higher perinatal adverse outcomes is also reported elsewhere in the literature (26, 27).

Compared to pregnancies in women aged 20–24, AMA was associated with all the adverse maternal and perinatal outcomes assessed (Figures 1, 2). Our study also indicates a direct association between adverse maternal outcomes and advancing maternal age categories. The MMR in the AMA group (291 deaths per 100,000 live births) was almost twice that of the entire cohort (145 deaths per 100,000 live births), four times that of adolescents, and three times that of pregnancies in women aged 20–24. This pattern is in keeping with the J-shaped age maternal age-related mortality curve, where mortality is acutely and persistently higher after age 30 (4, 28). These findings were consistent across the global network regions in our cohort (Africa 314, Southeast Asia 381, and Guatemala 201).

The current study has several limitations. Not all relevant confounders are collected in the global network registry, specifically medical conditions that could increase the risk of adverse pregnancy and maternal and perinatal outcomes. These morbidities are common in AMA, where we find the greatest association with these adverse outcomes in this study. Variables such as household income and marital status are important sociodemographic factors critical to assessing the risk of maternal and perinatal adverse outcomes. However, maternal education, which can serve as a proxy, was used in this study (29, 30). Data on the quality of hospital care is also unavailable and could provide insights into the divergence between our cohort's maternal and perinatal outcomes. Another study limitation is the exclusion of women who had a spontaneous abortion or other pregnancy loss that occurred <20 weeks, those with a medically terminated pregnancy at any point, and women who gave birth to infants with a birthweight of less than 500 g. These are all important adverse pregnancy outcomes that can affect maternal, perinatal, and neonatal outcomes. We did not include BMI in the regression model as it was unavailable for the Kenya cohort before 2017. We also do not have pre-pregnancy or early BMI for most women. BMI is a known predictor of adverse maternal outcomes and may explain some of our study findings. The global network registry collaborates closely with delivery units within the catchment areas to obtain accurate data. Given that about 75% of the deliveries were either in a hospital or health center, the documentation of crucial quality metrics (presence of a skilled health worker and use of skin-to-skin Kangaroo mother care) is robust. Another limitation of this study is that the generated data originates from a limited geographical area and may not represent the country, other LMICs, or facility-based settings. However, the population-based nature of the registry makes the findings more generalizable than facility-based studies (26). Another strength of the study is that it is one of the largest prospective population-based studies of maternal and perinatal data in LMICs. The global network registry follow-up rates (93%) were high for the proportion of subjects enrolled in the registry.

In conclusion, the MMR in this large cohort involving six LMICs is high compared to the 2030 sustainable development MMR target goal and varied by region and maternal age group. Adolescent pregnancy was associated with a lower adjusted risk of adverse maternal outcomes but an increased adjusted risk of adverse perinatal outcomes. The prevalence of AMA pregnancy is low but associated with a higher adjusted risk of maternal and perinatal adverse outcomes.

## Data Availability

The raw data supporting the conclusions of this article will be made available by the authors, without undue reservation.
