# Supplementary material for: Maternal age extremes and adverse pregnancy outcomes in low-resourced settings
Source: Front Glob Womens Health. 2023 Nov 28;4:1201037. doi: 10.3389/fgwh.2023.1201037 (PMC10715413; doi:10.3389/fgwh.2023.1201037)
Supplement: Supplementary file 1 [file Datasheet1.zip › Data Sheet 1_v1/Supplemental Figures Title and Footnote.docx]

|  |
| --- |
| **Supplemental Figure 1: Risk of adverse maternal outcomes by maternal age groups compared to mothers aged 20-24 for the African region** |

Relative risks obtained from a Poisson model adjusting for maternal age, site, parity, multiple gestation, maternal education (some or none), ANC care and delivery location.

|  |
| --- |
| **Supplemental Figure 2: Risk of perinatal and neonatal outcomes by maternal age groups compared to mothers aged 20-24 for the African region** |

Relative risks obtained from a Poisson model adjusting for maternal age, site, parity, multiple gestation, maternal education (some or none), ANC care and delivery location.

|  |
| --- |
| **Supplemental Figure 3: Risk of adverse maternal outcomes by maternal age groups compared to mothers aged 20-24 for the Asian region** |

Relative risks obtained from a Poisson model adjusting for maternal age, site, parity, multiple gestation, maternal education (some or none), ANC care and delivery location.

|  |
| --- |
| **Supplemental Figure 4: Risk of perinatal and neonatal outcomes by maternal age groups compared to mothers aged 20-24 for the Asian region** |

| Relative risks obtained from a Poisson model adjusting for maternal age, site, parity, multiple gestation, maternal education (some or none), ANC care and delivery location. |
| --- |
|  |

|  |
| --- |
| **Supplemental Figure 5: Risk of adverse maternal outcomes by maternal age groups compared to mothers aged 20-24 for Guatemala** |

Relative risks obtained from a Poisson model adjusting for maternal age, site, parity, multiple gestation, maternal education (some or none), ANC care and delivery location. The model for maternal mortality in Guatemala did not converge.

|  |
| --- |
| **Supplemental Figure 6: Risk of perinatal and neonatal outcomes by maternal age groups compared to mothers aged 20-24 for Guatemala** |

| Relative risks obtained from a Poisson model adjusting for maternal age, site, parity, multiple gestation, maternal education (some or none), ANC care and delivery location. |
| --- |
|  |

|  |
| --- |
| **Supplemental Figure 7: Risk of perinatal mortality** |

| Relative risks obtained from a Poisson model adjusting for maternal age, site, parity, multiple gestation, maternal education, ANC care, delivery location and the interactions between maternal age and education (P<0.001), ANC (P<0.001) and delivery location (P<0.001). |
| --- |

|  |
| --- |
| **Supplemental Figure 8: Risk of neonatal mortality < 28 days** |

| Relative risks obtained from a Poisson model adjusting for maternal age, site, parity, multiple gestation, maternal education, ANC care, delivery location and the interactions between maternal age and education (P=0.004), ANC (P<0.001) and delivery location (P<0.001). |
| --- |
