# Supplementary material for: Maternal age extremes and adverse pregnancy outcomes in low-resourced settings
Source: Front Glob Womens Health. 2023 Nov 28;4:1201037. doi: 10.3389/fgwh.2023.1201037 (PMC10715413; doi:10.3389/fgwh.2023.1201037)
Supplement: Supplementary file 1 [file Datasheet1.zip › Data Sheet 1_v1/Supplemental Tables Revised .docx]

|  |
| --- |
| **Supplemental Table 1: Maternal demographics and health care utilization by age group for the African region** |

|  |  | Maternal Age (years) | | | | | |
| --- | --- | --- | --- | --- | --- | --- | --- |
| Characteristic | Overall | < 18 | 18-19 | 20 - 24 | 25 - 29 | 30 - 35 | > 35 |
| Mothers, n (%) | 196,593 | 15,821 (8.0) | 28,236 (14.4) | 63,890 (32.5) | 43,140 (21.9) | 32,373 (16.5) | 13,133 (6.7) |
| Maternal education, n (%) | 196,409 | 15,805 | 28,217 | 63,830 | 43,102 | 32,344 | 13,111 |
| No formal schooling | 23,680 (12.1) | 702 (4.4) | 1,548 (5.5) | 5,257 (8.2) | 6,104 (14.2) | 6,709 (20.7) | 3,360 (25.6) |
| Primary/Secondary | 165,742 (84.4) | 15,012 (95.0) | 26,380 (93.5) | 56,244 (88.1) | 34,494 (80.0) | 24,235 (74.9) | 9,377 (71.5) |
| University + | 6,987 (3.6) | 91 (0.6) | 289 (1.0) | 2,329 (3.6) | 2,504 (5.8) | 1,400 (4.3) | 374 (2.9) |
| Parity, n (%) | 196,561 | 15,818 | 28,229 | 63,879 | 43,133 | 32,371 | 13,131 |
| 0 | 53,399 (27.2) | 14,620 (92.4) | 19,521 (69.2) | 16,074 (25.2) | 2,626 (6.1) | 465 (1.4) | 93 (0.7) |
| 1-2 | 73,032 (37.2) | 1,169 (7.4) | 8,426 (29.8) | 39,105 (61.2) | 18,300 (42.4) | 5,249 (16.2) | 783 (6.0) |
| 3 + | 70,130 (35.7) | 29 (0.2) | 282 (1.0) | 8,700 (13.6) | 22,207 (51.5) | 26,657 (82.3) | 12,255 (93.3) |
| Multiple birth, n (%) | 2,471 (1.3) | 81 (0.5) | 213 (0.8) | 659 (1.0) | 660 (1.5) | 621 (1.9) | 237 (1.8) |
| Body mass index (BMI) measured^1^ (Kg/m^2^), n (%) | 113,149 (99.9) | 9,977 (99.8) | 15,723 (99.9) | 33,377 (99.9) | 24,215 (99.9) | 20,407 (99.9) | 9,450 (99.8) |
| Mean (std) | 22.8 (3.4) | 21.8 (2.6) | 22.1 (2.6) | 22.6 (3.0) | 23.0 (3.6) | 23.4 (3.9) | 23.8 (4.2) |
| Median (min-max) | 22.3 (12 - 63) | 21.6 (13 - 63) | 21.9 (13 - 55) | 22.2 (12 - 50) | 22.5 (12 - 52) | 22.7 (12 - 57) | 23.0 (13 - 56) |
| At least one antenatal care (ANC) visit, n (%) | 193,507 (98.4) | 15,641 (98.9) | 27,915 (98.9) | 63,047 (98.7) | 42,421 (98.3) | 31,685 (97.9) | 12,798 (97.5) |
| At least four ANC visits, n/N (%) | 82,224/164,968 (49.8) | 6,733/13,060 (51.6) | 12,456/23,676 (52.6) | 27,496/53,399 (51.5) | 17,939/36,287 (49.4) | 12,739/27,468 (46.4) | 4,861/11,078 (43.9) |
| Trimester of first ANC visit, n (%) | 187,164 | 15,174 | 27,062 | 60,949 | 40,958 | 30,648 | 12,373 |
| First (0-14 wks) | 24,216 (12.9) | 2,407 (15.9) | 4,080 (15.1) | 8,487 (13.9) | 4,890 (11.9) | 3,141 (10.2) | 1,211 (9.8) |
| Second (15-28 wks) | 132,685 (70.9) | 10,818 (71.3) | 19,244 (71.1) | 42,809 (70.2) | 29,064 (71.0) | 21,918 (71.5) | 8,832 (71.4) |
| Third (29-42 wks) | 30,263 (16.2) | 1,949 (12.8) | 3,738 (13.8) | 9,653 (15.8) | 7,004 (17.1) | 5,589 (18.2) | 2,330 (18.8) |
| Delivery attendant, n (%) | 196,509 | 15,816 | 28,224 | 63,866 | 43,127 | 32,351 | 13,125 |
| Physician | 5,175 (2.6) | 470 (3.0) | 696 (2.5) | 1,521 (2.4) | 1,085 (2.5) | 920 (2.8) | 483 (3.7) |
| Nurse/Midwife/   Health worker | 131,935 (67.1) | 11,484 (72.6) | 19,727 (69.9) | 42,703 (66.9) | 28,576 (66.3) | 21,168 (65.4) | 8,277 (63.1) |
| Traditional birth   attendant | 40,845 (20.8) | 2,747 (17.4) | 5,667 (20.1) | 13,832 (21.7) | 9,171 (21.3) | 6,753 (20.9) | 2,675 (20.4) |
| Family/self/other | 18,554 (9.4) | 1,115 (7.0) | 2,134 (7.6) | 5,810 (9.1) | 4,295 (10.0) | 3,510 (10.8) | 1,690 (12.9) |
| Delivery location, n (%) | 196,516 | 15,818 | 28,225 | 63,869 | 43,127 | 32,352 | 13,125 |
| Hospital | 34,792 (17.7) | 3,259 (20.6) | 5,198 (18.4) | 11,151 (17.5) | 7,292 (16.9) | 5,423 (16.8) | 2,469 (18.8) |
| Clinic/Health center | 104,185 (53.0) | 8,813 (55.7) | 15,367 (54.4) | 33,311 (52.2) | 22,821 (52.9) | 17,317 (53.5) | 6,556 (50.0) |
| Home/Other | 57,539 (29.3) | 3,746 (23.7) | 7,660 (27.1) | 19,407 (30.4) | 13,014 (30.2) | 9,612 (29.7) | 4,100 (31.2) |
| Placed on mother's chest after delivery or skin to skin, n (%) | 145,896 (75.8) | 12,378 (80.2) | 21,420 (77.3) | 47,426 (75.6) | 31,593 (74.8) | 23,707 (75.0) | 9,372 (73.9) |

| ^1^ Kenya did not consistently obtain height measurements; therefore, Kenyan BMI data is excluded. |
| --- |

|  |
| --- |
| **Supplemental Table 2: Maternal and perinatal adverse outcomes by maternal age group for the African region** |

|  |  | Maternal Age (years) | | | | | |
| --- | --- | --- | --- | --- | --- | --- | --- |
| Characteristic | Overall | < 18 | 18-19 | 20 - 24 | 25 - 29 | 30 - 35 | > 35 |
| *Mothers, n* | 196,593 | 15,821 | 28,236 | 63,890 | 43,140 | 32,373 | 13,133 |
| Obstructed/prolonged labor/failure to progress, n (%) | 9,535 (4.9) | 1,002 (6.3) | 1,514 (5.4) | 2,911 (4.6) | 1,932 (4.5) | 1,514 (4.7) | 662 (5.0) |
| Antepartum hemorrhage, n (%) | 2,205 (1.1) | 199 (1.3) | 274 (1.0) | 620 (1.0) | 507 (1.2) | 418 (1.3) | 187 (1.4) |
| Postpartum hemorrhage, n (%) | 4,044 (2.1) | 312 (2.0) | 534 (1.9) | 1,294 (2.0) | 895 (2.1) | 722 (2.2) | 287 (2.2) |
| Evidence of hypertensive disease/severe pre-eclampsia/ eclampsia, n (%) | 1,664 (0.8) | 113 (0.7) | 203 (0.7) | 459 (0.7) | 350 (0.8) | 341 (1.1) | 198 (1.5) |
| Abnormal lie: breech, transverse, or oblique, n (%) | 2,032 (1.0) | 143 (0.9) | 267 (0.9) | 562 (0.9) | 443 (1.0) | 403 (1.2) | 214 (1.6) |
| Severe infection at follow-up, n (%) | 461 (0.3) | 40 (0.3) | 58 (0.2) | 140 (0.2) | 96 (0.2) | 84 (0.3) | 43 (0.4) |
| Cesarean delivery, n (%) | 3,313 (1.7) | 278 (1.8) | 432 (1.5) | 957 (1.5) | 717 (1.7) | 617 (1.9) | 312 (2.4) |
| Maternal death < 42 days, n (rate/100,000 live births) | 256 (132) | 14 (91) | 18 (65) | 56 (89) | 52 (122) | 75 (233) | 41 (319) |
| *Infants, N* | 199,156 | 15,904 | 28,453 | 64,564 | 43,818 | 33,027 | 13,390 |
| Stillbirth, n (rate/1000) | 4,812 (24.2) | 440 (27.7) | 602 (21.2) | 1,276 (19.8) | 1,086 (24.8) | 878 (26.6) | 530 (39.6) |
| Stillbirth type, n (%) | 4,693 | 427 | 584 | 1,240 | 1,063 | 861 | 518 |
| Macerated | 1,537 (32.8) | 104 (24.4) | 194 (33.2) | 397 (32.0) | 352 (33.1) | 302 (35.1) | 188 (36.3) |
| Fresh | 3,156 (67.2) | 323 (75.6) | 390 (66.8) | 843 (68.0) | 711 (66.9) | 559 (64.9) | 330 (63.7) |
| Neonatal death < 7 days, n (rate/1000) | 2,740 (14.2) | 311 (20.2) | 471 (17.0) | 757 (12.0) | 497 (11.7) | 470 (14.7) | 234 (18.3) |
| Neonatal death < 28 days, n (rate/1000) | 3,197 (16.5) | 367 (23.9) | 533 (19.2) | 875 (13.9) | 597 (14.0) | 553 (17.3) | 272 (21.3) |
| Perinatal mortality, n (rate/1000) | 7,552 (38.1) | 751 (47.5) | 1,073 (37.9) | 2,033 (31.6) | 1,583 (36.3) | 1,348 (41.0) | 764 (57.4) |
| Preterm birth, n (%) | 27,379 (14.1) | 2,994 (19.4) | 4,357 (15.8) | 8,073 (12.9) | 5,434 (12.7) | 4,451 (13.8) | 2,070 (15.8) |
| Low birth weight (< 2500g), n (%) | 14,219 (7.2) | 1,726 (10.9) | 2,376 (8.4) | 3,946 (6.1) | 2,676 (6.1) | 2,326 (7.1) | 1,169 (8.7) |

|  |
| --- |
| **Supplemental Table 3: Maternal demographics and health care utilization by age group for the Asian region** |

|  |  | Maternal Age (years) | | | | |
| --- | --- | --- | --- | --- | --- | --- |
| Characteristic | Overall | < 20 | 20 - 24 | 25 - 29 | 30 - 35 | > 35 |
| Mothers, n (%) | 315,087 | 19,202 (6.1) | 153,714 (48.8) | 98,961 (31.4) | 37,128 (11.8) | 6,082 (1.9) |
| Maternal education, n (%) | 314,321 | 19,141 | 153,288 | 98,729 | 37,088 | 6,075 |
| No formal schooling | 106,285 (33.8) | 4,176 (21.8) | 27,898 (18.2) | 42,173 (42.7) | 26,921 (72.6) | 5,117 (84.2) |
| Primary/secondary | 178,065 (56.7) | 14,024 (73.3) | 108,232 (70.6) | 46,877 (47.5) | 8,151 (22.0) | 781 (12.9) |
| University + | 29,971 (9.5) | 941 (4.9) | 17,158 (11.2) | 9,679 (9.8) | 2,016 (5.4) | 177 (2.9) |
| Parity, n (%) | 312,367 | 18,652 | 152,274 | 98,340 | 37,025 | 6,076 |
| 0 | 113,800 (36.4) | 17,360 (93.1) | 78,420 (51.5) | 15,658 (15.9) | 2,184 (5.9) | 178 (2.9) |
| 1-2 | 142,896 (45.7) | 1,255 (6.7) | 71,085 (46.7) | 59,750 (60.8) | 10,118 (27.3) | 688 (11.3) |
| 3 + | 55,671 (17.8) | 37 (0.2) | 2,769 (1.8) | 22,932 (23.3) | 24,723 (66.8) | 5,210 (85.7) |
| Multiple birth, n (%) | 2,776 (0.9) | 101 (0.5) | 1,122 (0.7) | 944 (1.0) | 505 (1.4) | 104 (1.7) |
| Body mass index (BMI) measured^1^ (Kg/m^2^), n (%) | 309,090 (98.1) | 18,568 (96.7) | 150,208 (97.7) | 97,389 (98.4) | 36,877 (99.3) | 6,048 (99.4) |
| Mean (std) | 20.3 (3.2) | 19.6 (2.8) | 19.9 (2.9) | 20.6 (3.3) | 21.4 (3.9) | 21.9 (4.2) |
| Median (min-max) | 20.0 (12 - 65) | 19.4 (12 - 59) | 19.6 (12 - 62) | 20.1 (12 - 60) | 20.8 (12 - 60) | 21.1 (13 - 65) |
| At least one antenatal care (ANC) visit, n (%) | 303,488 (96.4) | 18,961 (98.8) | 151,410 (98.6) | 94,179 (95.3) | 33,584 (90.5) | 5,354 (88.1) |
| At least four ANC visits, n/N (%) | 147,692/236,318 (62.5) | 9,492/13,971 (67.9) | 78,857/111,114 (71.0) | 44,973/76,584 (58.7) | 12,681/29,773 (42.6) | 1,689/4,876 (34.6) |
| Trimester of first ANC visit, n (%) | 273,315 | 17,604 | 141,403 | 82,939 | 27,164 | 4,205 |
| First (0-14 wks) | 181,669 (66.5) | 13,189 (74.9) | 103,132 (72.9) | 51,549 (62.2) | 12,305 (45.3) | 1,494 (35.5) |
| Second (15-28 wks) | 67,800 (24.8) | 3,555 (20.2) | 30,490 (21.6) | 22,488 (27.1) | 9,636 (35.5) | 1,631 (38.8) |
| Third (29-42 wks) | 23,846 (8.7) | 860 (4.9) | 7,781 (5.5) | 8,902 (10.7) | 5,223 (19.2) | 1,080 (25.7) |
| Delivery attendant, n (%) | 314,897 | 19,196 | 153,648 | 98,894 | 37,082 | 6,077 |
| Physician | 158,216 (50.2) | 10,806 (56.3) | 87,992 (57.3) | 45,386 (45.9) | 12,290 (33.1) | 1,742 (28.7) |
| Nurse/Midwife/   Health worker | 99,402 (31.6) | 6,452 (33.6) | 52,415 (34.1) | 30,258 (30.6) | 8,876 (23.9) | 1,401 (23.1) |
| Traditional birth   attendant | 45,107 (14.3) | 1,465 (7.6) | 9,432 (6.1) | 18,375 (18.6) | 13,259 (35.8) | 2,576 (42.4) |
| Family/self/other | 12,172 (3.9) | 473 (2.5) | 3,809 (2.5) | 4,875 (4.9) | 2,657 (7.2) | 358 (5.9) |
| Delivery location, n (%) | 314,883 | 19,188 | 153,631 | 98,896 | 37,092 | 6,076 |
| Hospital | 180,519 (57.3) | 12,891 (67.2) | 99,420 (64.7) | 50,910 (51.5) | 14,982 (40.4) | 2,316 (38.1) |
| Clinic/Health center | 87,587 (27.8) | 4,811 (25.1) | 42,453 (27.6) | 29,136 (29.5) | 9,722 (26.2) | 1,465 (24.1) |
| Home/Other | 46,777 (14.9) | 1,486 (7.7) | 11,758 (7.7) | 18,850 (19.1) | 12,388 (33.4) | 2,295 (37.8) |
| Placed on mother's chest after delivery or skin to skin, n (%) | 131,589 (42.9) | 8,250 (44.3) | 78,472 (52.3) | 36,904 (38.2) | 7,118 (19.8) | 845 (14.5) |

|  |
| --- |
| **Supplemental Table 4: Maternal and perinatal adverse outcomes by maternal age group for the Asian region** |

|  |  | Maternal Age (years) | | | | |
| --- | --- | --- | --- | --- | --- | --- |
| Characteristic | Overall | < 20 | 20 - 24 | 25 - 29 | 30 - 35 | > 35 |
| *Mothers, n* | 315,087 | 19,202 | 153,714 | 98,961 | 37,128 | 6,082 |
| Obstructed/prolonged labor/failure to progress, n (%) | 34,202 (10.9) | 2,529 (13.2) | 16,959 (11.0) | 9,921 (10.0) | 4,086 (11.0) | 707 (11.6) |
| Antepartum hemorrhage, n (%) | 4,550 (1.4) | 234 (1.2) | 1,280 (0.8) | 1,592 (1.6) | 1,136 (3.1) | 308 (5.1) |
| Postpartum hemorrhage, n (%) | 5,374 (1.7) | 215 (1.1) | 1,373 (0.9) | 2,003 (2.0) | 1,425 (3.9) | 358 (5.9) |
| Evidence of hypertensive disease/severe pre-eclampsia/ eclampsia, n (%) | 10,574 (3.4) | 673 (3.5) | 4,354 (2.8) | 3,168 (3.2) | 1,956 (5.3) | 423 (7.0) |
| Abnormal lie: breech, transverse, or oblique, n (%) | 7,313 (2.3) | 436 (2.3) | 3,205 (2.1) | 2,277 (2.3) | 1,160 (3.1) | 235 (3.9) |
| Severe infection at follow-up, n (%) | 1,773 (0.6) | 133 (0.7) | 546 (0.4) | 571 (0.6) | 397 (1.2) | 126 (2.2) |
| Cesarean delivery, n (%) | 56,977 (18.1) | 3,350 (17.4) | 29,733 (19.4) | 17,751 (17.9) | 5,443 (14.7) | 700 (11.5) |
| Maternal death < 42 days, n (rate/100,000 live births) | 528 (172) | 21 (112) | 194 (129) | 183 (189) | 107 (300) | 23 (399) |
| *Infants, N* | 317,916 | 19,306 | 154,851 | 99,923 | 37,649 | 6,187 |
| Stillbirth, n (rate/1000) | 10,204 (32.1) | 577 (29.9) | 3,998 (25.8) | 3,277 (32.8) | 1,927 (51.2) | 425 (68.7) |
| Stillbirth type, n (%) | 9,260 | 548 | 3,711 | 2,919 | 1,702 | 380 |
| Macerated | 3,097 (33.4) | 181 (33.0) | 1,192 (32.1) | 964 (33.0) | 618 (36.3) | 142 (37.4) |
| Fresh | 6,163 (66.6) | 367 (67.0) | 2,519 (67.9) | 1,955 (67.0) | 1,084 (63.7) | 238 (62.6) |
| Neonatal death < 7 days, n (rate/1000) | 7,398 (24.1) | 494 (26.4) | 3,138 (20.8) | 2,306 (24.0) | 1,217 (34.3) | 243 (42.5) |
| Neonatal death < 28 days, n (rate/1000) | 9,234 (30.1) | 587 (31.4) | 3,861 (25.7) | 2,908 (30.2) | 1,564 (44.1) | 314 (54.9) |
| Perinatal mortality, n (rate/1000) | 17,602 (55.6) | 1,071 (55.6) | 7,136 (46.2) | 5,583 (56.1) | 3,144 (84.0) | 668 (108.7) |
| Preterm birth, n (%) | 43,860 (14.2) | 2,658 (14.0) | 18,479 (12.2) | 14,537 (15.0) | 6,980 (19.2) | 1,206 (20.4) |
| Low birth weight (< 2500g), n (%) | 59,637 (18.8) | 4,377 (22.7) | 28,659 (18.6) | 17,876 (18.0) | 7,411 (19.8) | 1,314 (21.4) |

|  |
| --- |
| **Supplemental Table 5: Maternal demographics and health care utilization by age group for Guatemala** |

|  |  | Maternal Age (years) | | | | | |
| --- | --- | --- | --- | --- | --- | --- | --- |
| Characteristic | Overall | < 18 | 18-19 | 20 - 24 | 25 - 29 | 30 - 35 | > 35 |
| Mothers, n (%) | 91,204 | 6,546 (7.2) | 8,779 (9.6) | 27,685 (30.4) | 21,482 (23.6) | 17,250 (18.9) | 9,462 (10.4) |
| Maternal education, n (%) | 91,195 | 6,545 | 8,778 | 27,684 | 21,478 | 17,249 | 9,461 |
| No formal schooling | 13,131 (14.4) | 483 (7.4) | 622 (7.1) | 2,856 (10.3) | 2,987 (13.9) | 3,468 (20.1) | 2,715 (28.7) |
| Primary/secondary | 73,392 (80.5) | 6,032 (92.2) | 7,871 (89.7) | 23,203 (83.8) | 16,940 (78.9) | 12,825 (74.4) | 6,521 (68.9) |
| University + | 4,672 (5.1) | 30 (0.5) | 285 (3.2) | 1,625 (5.9) | 1,551 (7.2) | 956 (5.5) | 225 (2.4) |
| Parity, n (%) | 91,200 | 6,545 | 8,779 | 27,684 | 21,480 | 17,250 | 9,462 |
| 0 | 26,741 (29.3) | 5,751 (87.9) | 6,016 (68.5) | 10,408 (37.6) | 3,339 (15.5) | 1,038 (6.0) | 189 (2.0) |
| 1-2 | 35,419 (38.8) | 788 (12.0) | 2,723 (31.0) | 14,904 (53.8) | 10,683 (49.7) | 5,201 (30.2) | 1,120 (11.8) |
| 3 + | 29,040 (31.8) | 6 (0.1) | 40 (0.5) | 2,372 (8.6) | 7,458 (34.7) | 11,011 (63.8) | 8,153 (86.2) |
| Multiple birth, n (%) | 625 (0.7) | 24 (0.4) | 24 (0.3) | 148 (0.5) | 147 (0.7) | 185 (1.1) | 97 (1.0) |
| Body mass index (BMI) measured^1^ (Kg/m^2^), n (%) | 72,921 (80.0) | 5,155 (78.8) | 6,953 (79.2) | 22,531 (81.4) | 17,482 (81.4) | 13,573 (78.7) | 7,227 (76.4) |
| Mean (std) | 26.2 (4.0) | 24.0 (3.0) | 24.5 (3.3) | 25.4 (3.6) | 26.6 (3.9) | 27.6 (4.2) | 28.1 (4.3) |
| Median (min-max) | 25.7 (14 - 64) | 23.8 (14 - 47) | 24.2 (14 - 46) | 25.0 (14 - 64) | 26.2 (14 - 59) | 27.2 (14 - 56) | 27.6 (14 - 54) |
| At least one antenatal care (ANC) visit, n (%) | 88,034 (96.5) | 6,373 (97.4) | 8,528 (97.2) | 26,833 (96.9) | 20,770 (96.7) | 16,569 (96.1) | 8,961 (94.7) |
| At least four ANC visits, n/N (%) | 53,829/81,634 (65.9) | 3,919/5,909 (66.3) | 5,287/7,876 (67.1) | 16,836/24,818 (67.8) | 13,056/19,253 (67.8) | 9,844/15,354 (64.1) | 4,887/8,424 (58.0) |
| Trimester of first ANC visit, n (%) | 83,623 | 6,106 | 8,151 | 25,579 | 19,750 | 15,667 | 8,370 |
| First (0-14 wks) | 35,263 (42.2) | 2,644 (43.3) | 3,577 (43.9) | 11,326 (44.3) | 8,800 (44.6) | 6,371 (40.7) | 2,545 (30.4) |
| Second (15-28 wks) | 40,258 (48.1) | 2,947 (48.3) | 3,915 (48.0) | 12,059 (47.1) | 9,182 (46.5) | 7,584 (48.4) | 4,571 (54.6) |
| Third (29-42 wks) | 8,102 (9.7) | 515 (8.4) | 659 (8.1) | 2,194 (8.6) | 1,768 (9.0) | 1,712 (10.9) | 1,254 (15.0) |
| Delivery attendant, n (%) | 91,193 | 6,545 | 8,779 | 27,684 | 21,480 | 17,245 | 9,460 |
| Physician | 49,232 (54.0) | 4,155 (63.5) | 5,215 (59.4) | 15,298 (55.3) | 11,221 (52.2) | 8,709 (50.5) | 4,634 (49.0) |
| Nurse/Midwife/   Health worker | 982 (1.1) | 81 (1.2) | 102 (1.2) | 298 (1.1) | 211 (1.0) | 187 (1.1) | 103 (1.1) |
| Traditional birth   attendant | 40,550 (44.5) | 2,269 (34.7) | 3,431 (39.1) | 11,982 (43.3) | 9,941 (46.3) | 8,271 (48.0) | 4,656 (49.2) |
| Family/self/other | 429 (0.5) | 40 (0.6) | 31 (0.4) | 106 (0.4) | 107 (0.5) | 78 (0.5) | 67 (0.7) |
| Delivery location, n (%) | 91,193 | 6,545 | 8,779 | 27,684 | 21,480 | 17,245 | 9,460 |
| Hospital | 46,155 (50.6) | 3,979 (60.8) | 4,989 (56.8) | 14,475 (52.3) | 10,392 (48.4) | 7,989 (46.3) | 4,331 (45.8) |
| Clinic/Health center | 1,820 (2.0) | 127 (1.9) | 151 (1.7) | 502 (1.8) | 475 (2.2) | 389 (2.3) | 176 (1.9) |
| Home/Other | 43,218 (47.4) | 2,439 (37.3) | 3,639 (41.5) | 12,707 (45.9) | 10,613 (49.4) | 8,867 (51.4) | 4,953 (52.4) |
| Placed on mother's chest after delivery or skin to skin, n (%) | 35,641 (40.1) | 2,948 (46.3) | 3,746 (43.6) | 11,096 (40.9) | 8,044 (38.3) | 6,361 (38.0) | 3,446 (37.9) |

|  |
| --- |
| **Supplemental Table 6: Maternal and perinatal adverse outcomes by maternal age group for Guatemala** |

|  |  | Maternal Age (years) | | | | | |
| --- | --- | --- | --- | --- | --- | --- | --- |
| Characteristic | Overall | < 18 | 18-19 | 20 - 24 | 25 - 29 | 30 - 35 | > 35 |
| *Mothers, n* | 91,204 | 6,546 | 8,779 | 27,685 | 21,482 | 17,250 | 9,462 |
| Obstructed/prolonged labor/failure to progress, n (%) | 5,439 (6.0) | 519 (7.9) | 634 (7.2) | 1,765 (6.4) | 1,124 (5.2) | 885 (5.1) | 512 (5.4) |
| Antepartum hemorrhage, n (%) | 663 (0.7) | 43 (0.7) | 49 (0.6) | 177 (0.6) | 148 (0.7) | 147 (0.9) | 99 (1.0) |
| Postpartum hemorrhage, n (%) | 1,481 (1.6) | 93 (1.4) | 123 (1.4) | 431 (1.6) | 354 (1.6) | 285 (1.7) | 195 (2.1) |
| Evidence of hypertensive disease/severe pre-eclampsia/ eclampsia, n (%) | 3,750 (4.1) | 241 (3.7) | 295 (3.4) | 981 (3.5) | 786 (3.7) | 813 (4.7) | 634 (6.7) |
| Abnormal lie: breech, transverse, or oblique, n (%) | 2,949 (3.2) | 184 (2.8) | 242 (2.8) | 767 (2.8) | 689 (3.2) | 642 (3.7) | 425 (4.5) |
| Severe infection at follow-up, n (%) | 380 (0.4) | 32 (0.5) | 31 (0.4) | 94 (0.3) | 95 (0.4) | 82 (0.5) | 46 (0.5) |
| Cesarean delivery, n (%) | 23,390 (25.6) | 1,621 (24.8) | 2,285 (26.0) | 7,362 (26.6) | 5,756 (26.8) | 4,265 (24.7) | 2,101 (22.2) |
| Maternal death < 42 days, n (rate/100,000 live births) | 88 (98) | 3 (47) | 8 (92) | 19 (69) | 15 (70) | 24 (141) | 19 (206) |
| *Infants, N* | 91,846 | 6,571 | 8,804 | 27,835 | 21,634 | 17,439 | 9,563 |
| Stillbirth, n (rate/1000) | 1,726 (18.8) | 130 (19.8) | 120 (13.6) | 385 (13.8) | 346 (16.0) | 393 (22.5) | 352 (36.8) |
| Stillbirth type, n (%) | 1,636 | 120 | 113 | 364 | 315 | 379 | 345 |
| Macerated | 350 (21.4) | 27 (22.5) | 22 (19.5) | 67 (18.4) | 66 (21.0) | 85 (22.4) | 83 (24.1) |
| Fresh | 1,286 (78.6) | 93 (77.5) | 91 (80.5) | 297 (81.6) | 249 (79.0) | 294 (77.6) | 262 (75.9) |
| Neonatal death < 7 days, n (rate/1000) | 1,418 (15.8) | 97 (15.1) | 123 (14.2) | 388 (14.2) | 290 (13.7) | 308 (18.1) | 212 (23.1) |
| Neonatal death < 28 days, n (rate/1000) | 2,096 (23.3) | 146 (22.7) | 193 (22.3) | 567 (20.7) | 418 (19.7) | 443 (26.0) | 329 (35.8) |
| Perinatal mortality, n (rate/1000) | 3,144 (34.3) | 227 (34.6) | 243 (27.7) | 773 (27.8) | 636 (29.5) | 701 (40.3) | 564 (59.1) |
| Preterm birth, n (%) | 9,866 (11.0) | 923 (14.4) | 1,038 (12.1) | 2,952 (10.8) | 2,071 (9.8) | 1,777 (10.4) | 1,105 (11.8) |
| Low birth weight (< 2500g), n (%) | 14,660 (16.0) | 1,294 (19.7) | 1,547 (17.6) | 4,374 (15.7) | 3,116 (14.4) | 2,726 (15.7) | 1,603 (16.8) |
