# Supplementary figures and images for: Maternal age extremes and adverse pregnancy outcomes in low-resourced settings
Source: Front Glob Womens Health. 2023 Nov 28;4:1201037. doi: 10.3389/fgwh.2023.1201037 (PMC10715413; doi:10.3389/fgwh.2023.1201037)

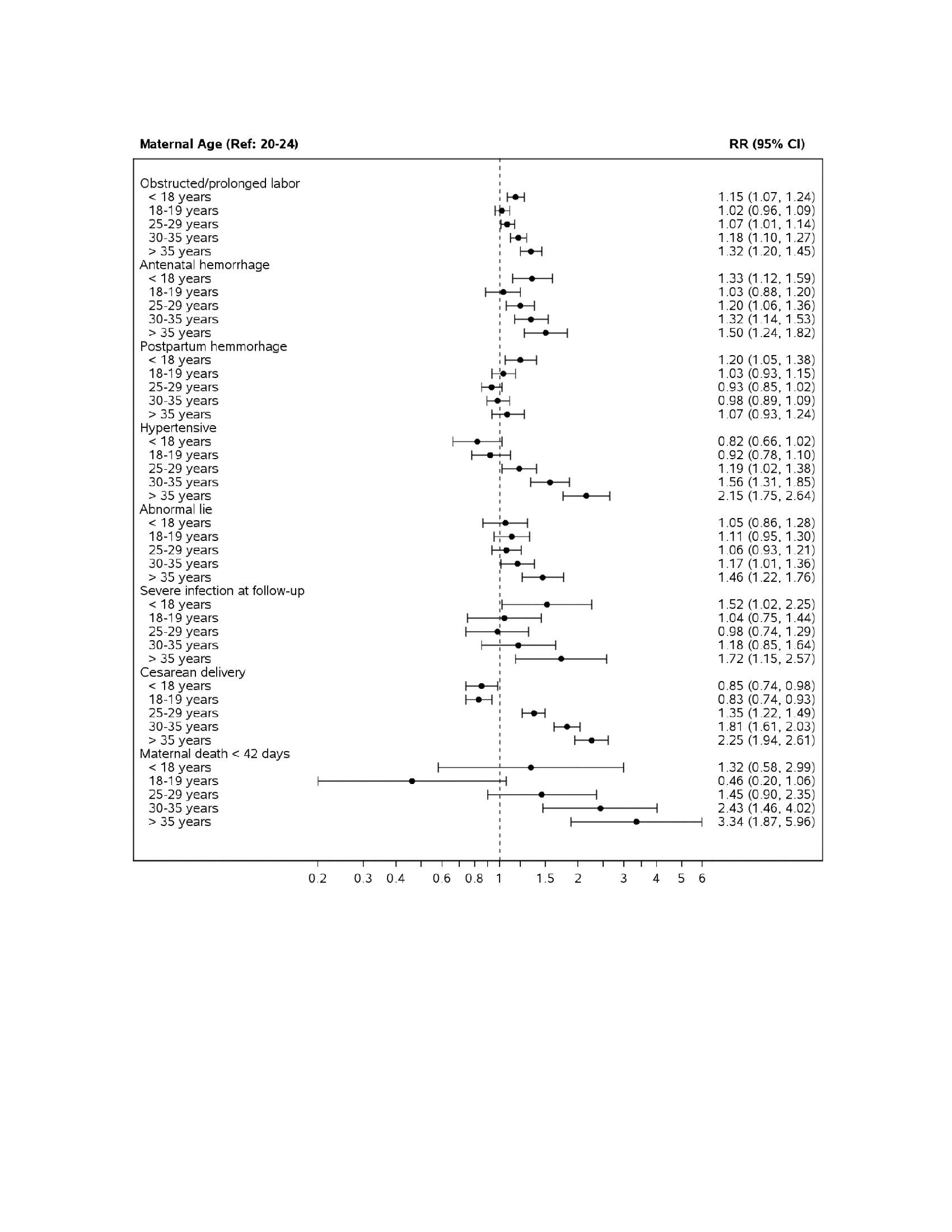

Supplement: Supplementary file 1 [file Datasheet1.zip › Data Sheet 1_v1/Supp-Figure-1.tiff]

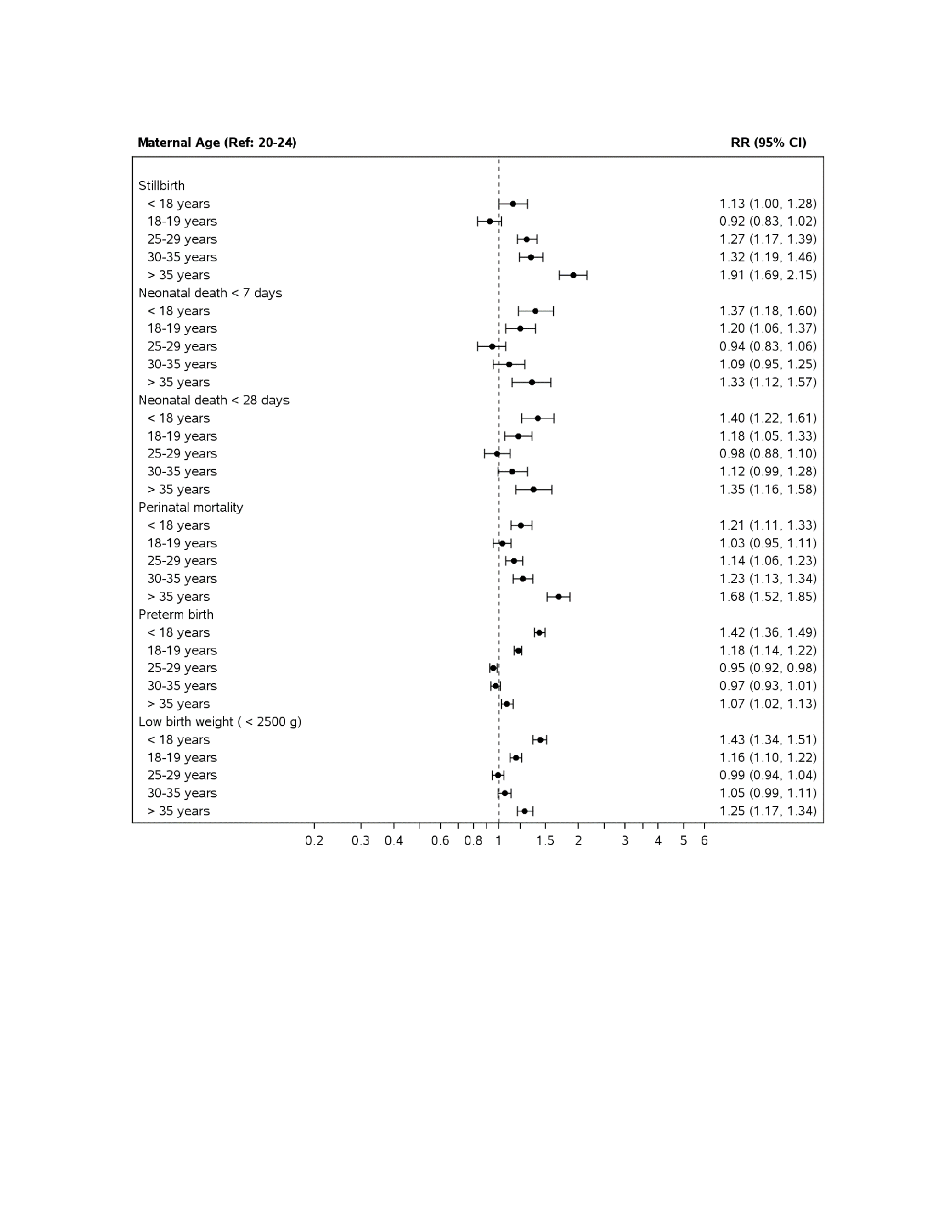

Supplement: Supplementary file 1 [file Datasheet1.zip › Data Sheet 1_v1/Supp-Figure-2.tiff]

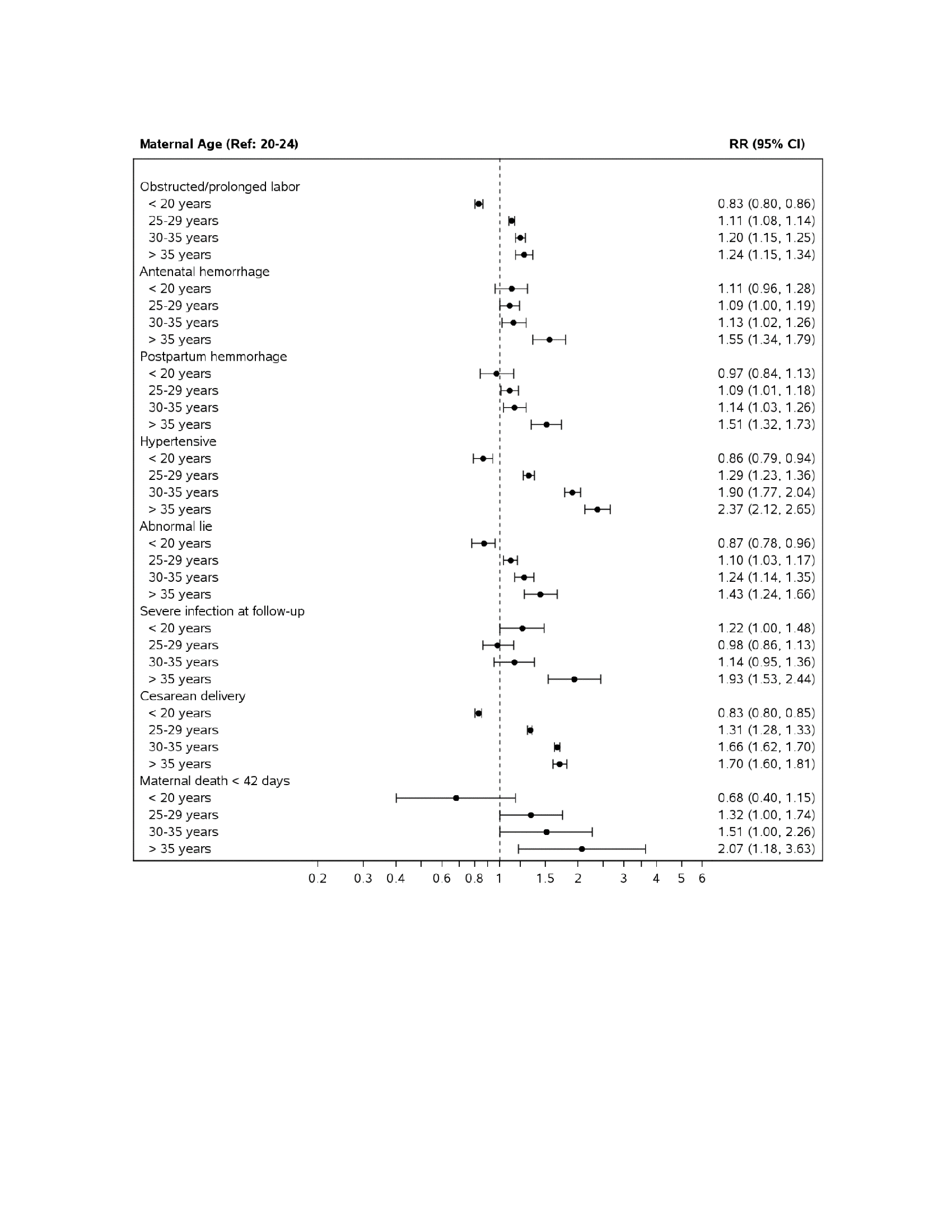

Supplement: Supplementary file 1 [file Datasheet1.zip › Data Sheet 1_v1/Supp-Figure-3.tiff]

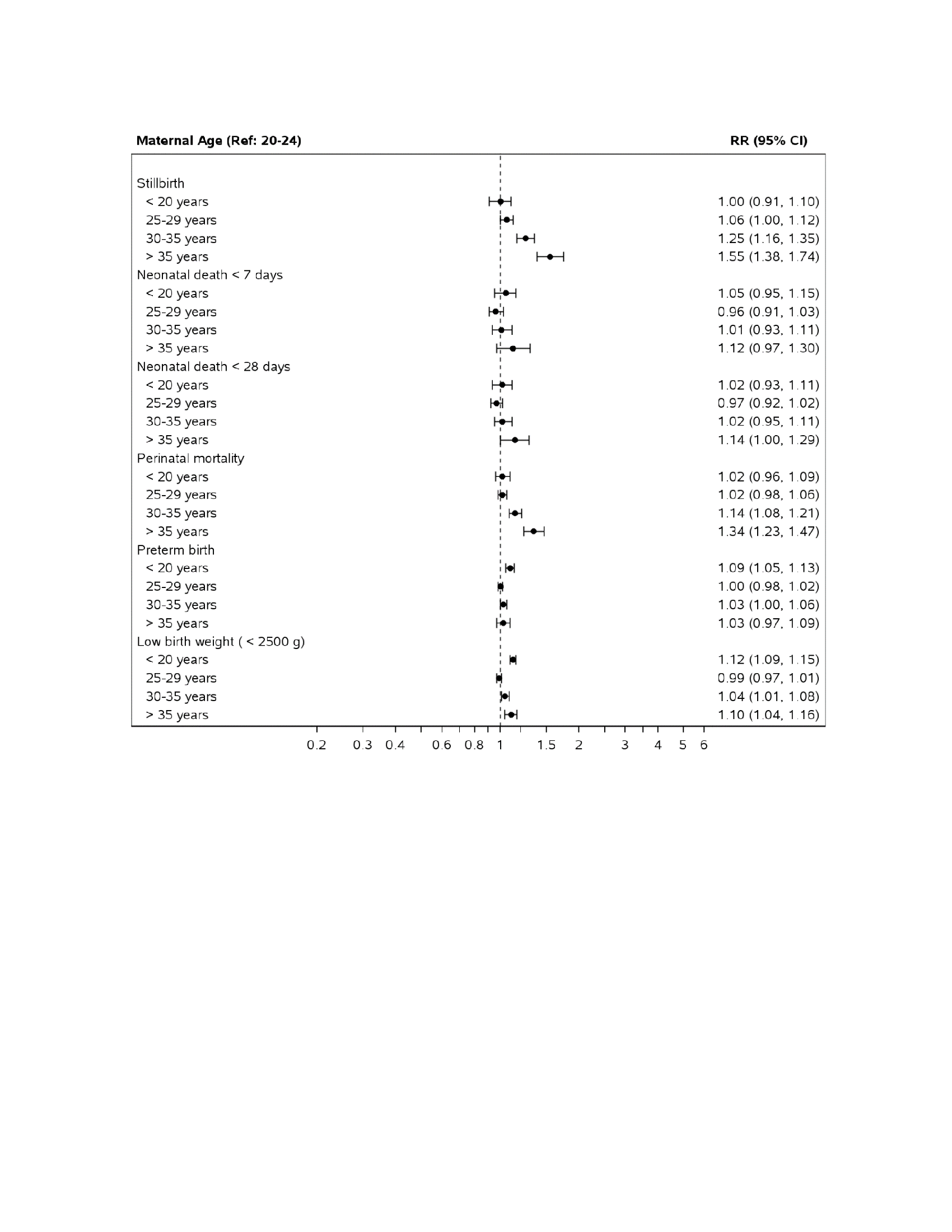

Supplement: Supplementary file 1 [file Datasheet1.zip › Data Sheet 1_v1/Supp-Figure-4.tiff]

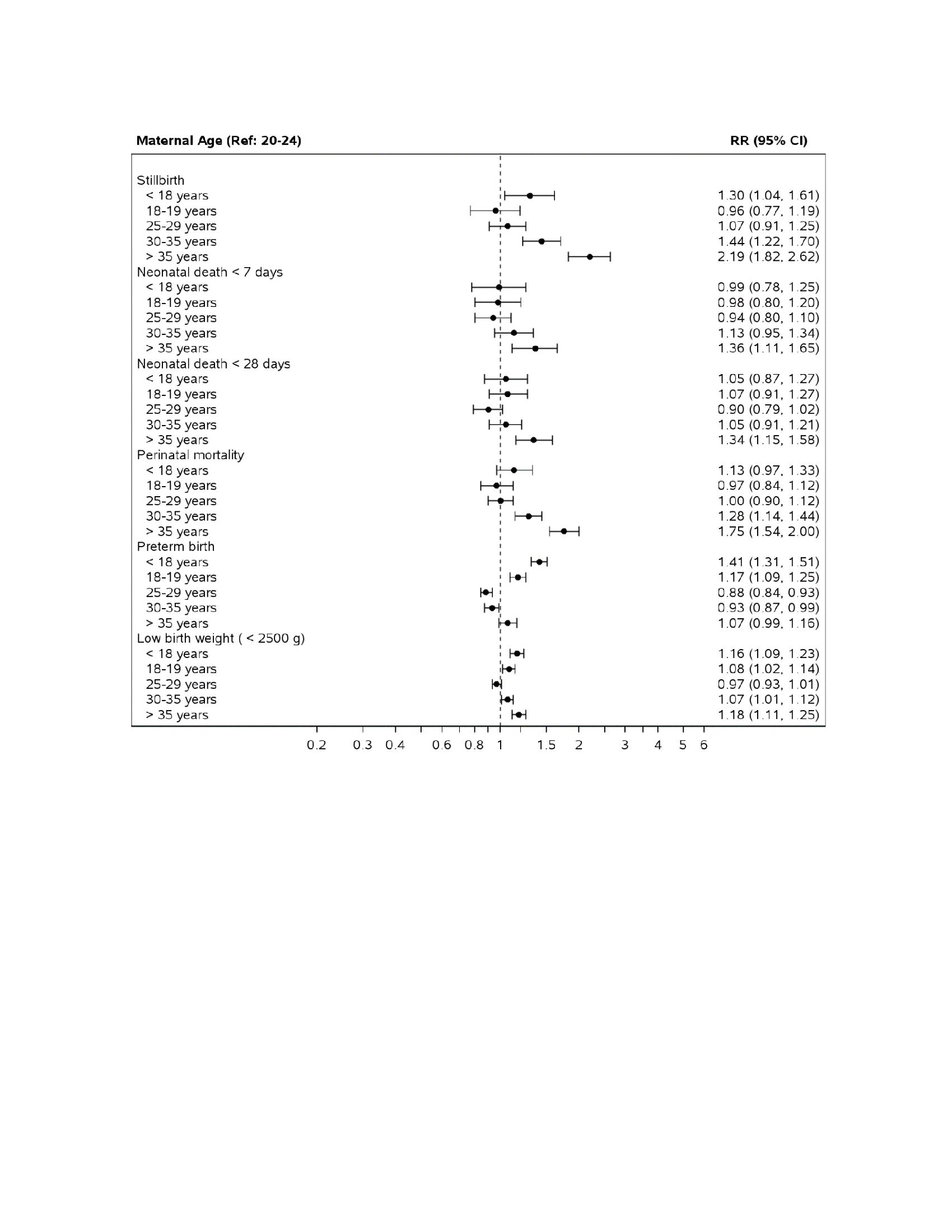

Supplement: Supplementary file 1 [file Datasheet1.zip › Data Sheet 1_v1/Supp-Figure-6.tiff]

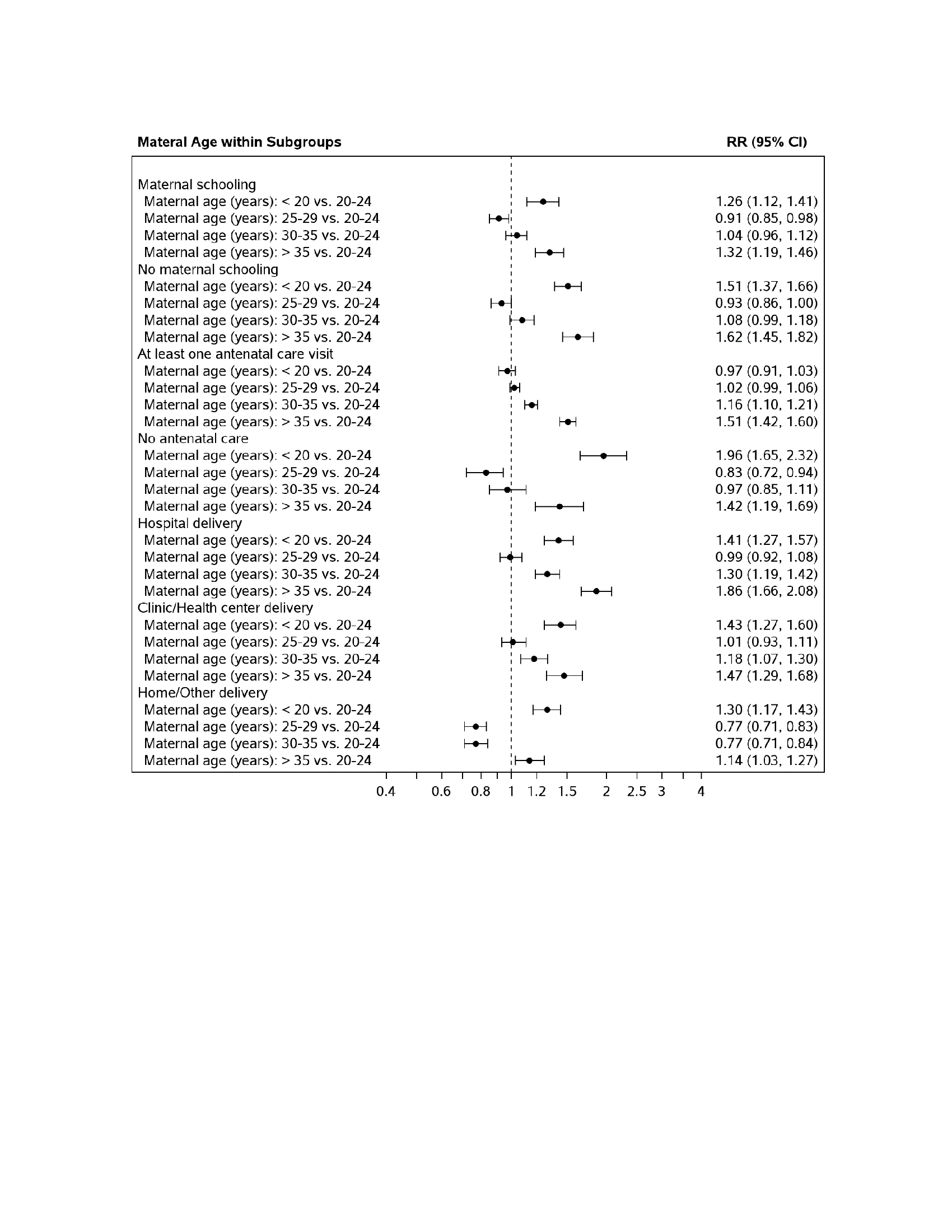

Supplement: Supplementary file 1 [file Datasheet1.zip › Data Sheet 1_v1/Supp-Figure-7.tiff]

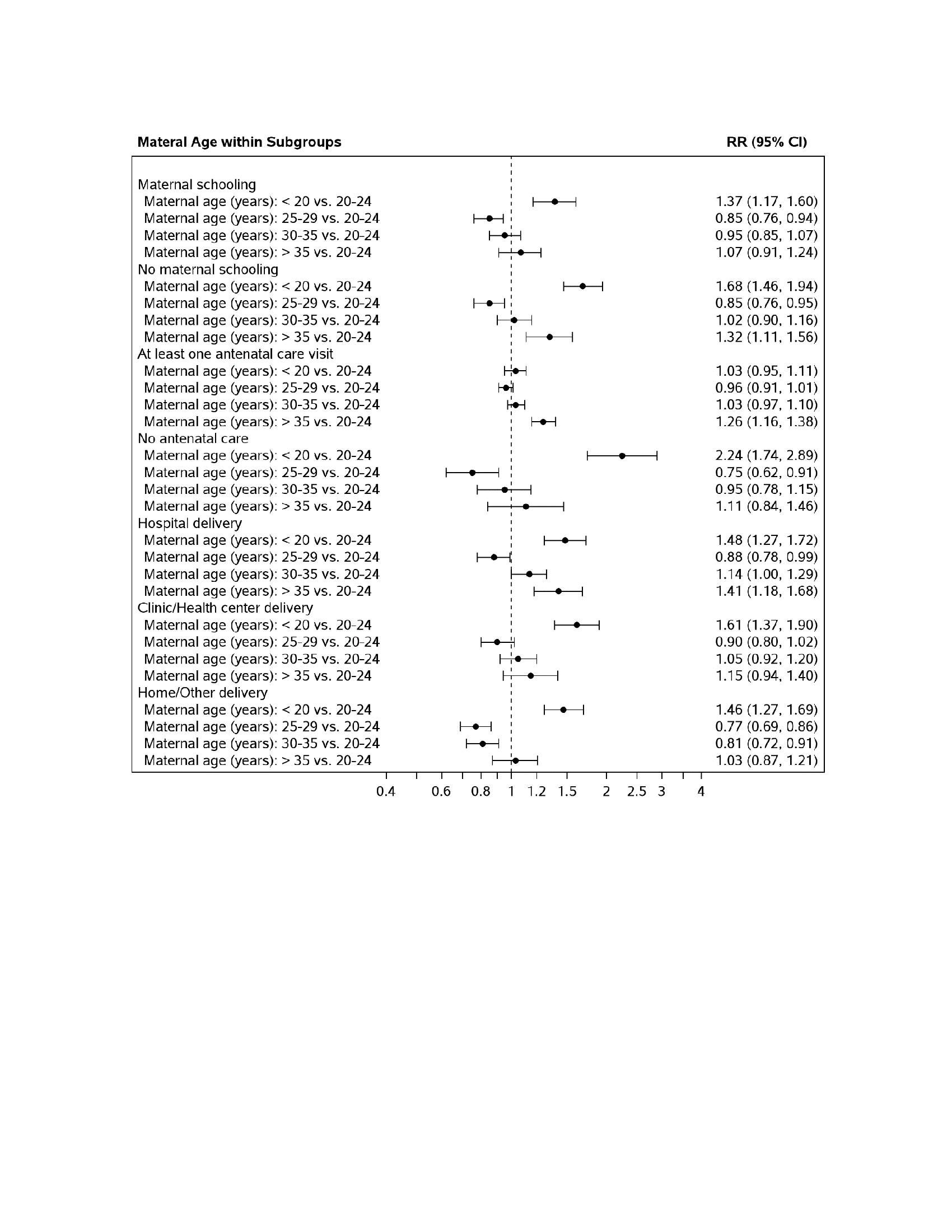

Supplement: Supplementary file 1 [file Datasheet1.zip › Data Sheet 1_v1/Supp-Figure-8.tiff]
